# Drip Fertigation Optimizes the Spatial Distribution and Translocation of Nitrogen, Thereby Increasing Yields and Improving Water and Nitrogen Use Efficiency in High-Density Summer Maize

**DOI:** 10.3390/plants15132026

**Published:** 2026-06-30

**Authors:** Chenxi Liu, Dong Cui, Xingyuan Chen, Shuo Cheng, Baizhao Ren, Ningning Yu, Jiwang Zhang

**Affiliations:** College of Agronomy, Shandong Agricultural University, Taian 271018, China; 2024120044@sdau.edu.cn (C.L.); cuidong4129@163.com (D.C.); chenxingyanstudy@foxmail.com (X.C.); a2785122173@163.com (S.C.); renbaizhao@sina.com (B.R.)

**Keywords:** summer maize, drip fertigation, planting density, nitrogen accumulation and translocation, water and nitrogen use efficiency

## Abstract

Achieving simultaneous improvements in grain yield and water–nitrogen use efficiency remains a major challenge in high-density summer maize production. Therefore, this study investigated how drip fertigation (DI) regulates soil nitrogen spatial distribution, plant nitrogen translocation, and ultimately resource use efficiency. A two-year field experiment (2023–2024) was conducted in Tai’an, Shandong, China, using a split-plot design. Two water–fertilizer management regimes, conventional border irrigation (BI) and drip fertigation (DI), were assigned to the main plots, while eight planting densities (15,000–120,000 plants ha^−1^) were allocated to the subplots. Two summer maize cultivars, Denghai 605 (DH605) and MY73, were evaluated. Compared with BI, DI significantly increased grain yield as well as water and nitrogen use efficiency. For DH605 and MY73, grain yield increased by 7.3% and 3.8%, respectively, accompanied by increases of 18.4% and 16.3% in WUE and 7.4% and 3.5% in NPFP. DI enhanced nitrogen accumulation within the 0–20 cm root zone while reducing nitrate-N residues in the 20–60 cm soil layer, thereby improving the spatial distribution and availability of root-zone nitrogen. Consequently, DI increased nitrogen translocation from vegetative organs to grains, as reflected by higher NTA, NTE, and NHI values, which promoted grain nitrogen accumulation and improved nitrogen use efficiency. Notably, DI did not significantly affect nitrogen uptake efficiency (NU_p_E), suggesting that the improvement in nitrogen utilization efficiency (NU_t_E) was driven primarily by enhanced nitrogen remobilization from vegetative organs to grains rather than by increased nitrogen uptake. Overall, drip fertigation improved grain yield, water use efficiency, and nitrogen use efficiency in high-density summer maize by optimizing root-zone nitrogen availability and promoting post-silking nitrogen translocation to grains.

## 1. Introduction

Securing global food supplies while improving the efficiency of water and nutrient use remains a major challenge for sustainable agricultural development [1]. Maize, one of the most important cereal crops worldwide, plays a vital role in ensuring food security, supporting livestock production, and maintaining the stability of agricultural markets [2,3]. However, maize production relies heavily on irrigation and synthetic fertilizers, and the conventional high-input production system is increasingly constrained by escalating resource consumption and environmental concerns [4]. Therefore, developing precision management strategies that simultaneously enhance productivity, improve resource use efficiency, and reduce environmental impacts has become a key objective of modern maize production.

Among agronomic management practices, water and fertilizer management are major determinants of maize productivity and resource use efficiency. Conventional summer maize production typically relies on border or flood irrigation combined with a single basal fertilizer application. Such an extensive management strategy often results in a temporal mismatch between water–nitrogen supply and crop demand. During the seedling stage, excessive irrigation and fertilization frequently lead to water percolation and nutrient losses, whereas during periods of peak demand in the mid-to-late growth stages, soil nutrient and water supply may become insufficient, accelerating leaf senescence and limiting yield formation [5,6]. In addition, nitrogen losses not only reduce fertilizer-use efficiency and increase production costs but also contribute to environmental problems through nitrate leaching and gaseous emissions [7,8]. By contrast, drip fertigation (DI) supplies water and nutrients directly to the root zone in a precise and split-application manner, thereby improving the synchronization between resource supply and crop demand while promoting the accumulation of water and nutrients within the active root zone [2,9]. Numerous studies have demonstrated that DI enhances water and nitrogen use efficiency, reduces nutrient movement to deeper soil layers, and mitigates environmental risks while maintaining or increasing crop yield [10,11,12]. This improvement is largely attributed to the enhanced synchronization between water–nitrogen supply and crop demand in the root zone, which reduces nitrogen losses through leaching and volatilization while improving nitrogen availability during critical growth stages [13]. However, previous studies have mainly focused on the agronomic benefits of DI in terms of yield improvement and resource use efficiency. Whether DI can further enhance yield potential and nitrogen use efficiency in high-density maize populations by regulating root-zone nitrogen distribution and plant nitrogen remobilization remains largely unclear.

Beyond water–fertilizer management, planting density is another critical factor influencing maize population yield. Increasing planting density is one of the most effective and economical agronomic strategies to maximize light interception and achieve yield gains [14,15,16]. However, when density exceeds the level that a given variety and soil fertility can sustain, competition among individual plants for light, water, and nutrients intensifies sharply. This often reduces per-plant productivity, raises lodging risk, and can exacerbate imbalances between water and nutrient supply and demand, ultimately lowering resource use efficiency [17]. Under high planting density, root overlap among neighboring plants increases, intensifying competition for soil water and nitrogen resources [15]. This may reduce nitrogen availability per plant and constrain crop growth and productivity. Such intensified belowground competition can alter nitrogen uptake dynamics and accelerate the limitation of soil nitrogen availability per plant, thereby affecting both biomass accumulation and nitrogen remobilization efficiency during reproductive stages [15,18]. The concept of “optimal planting density” therefore lies in finding a dynamic balance between yield improvement and resource competition. The optimum is not fixed but is influenced by variety characteristics, soil fertility, and water–fertilizer management practices [19,20]. Optimizing water and fertilizer management can alleviate resource competition under dense planting, helping maintain productivity stability and promoting simultaneous increases in yield and resource use efficiency. Particularly under high-density cultivation, adequate root-zone nitrogen supply and the plant’s capacity to remobilize nitrogen during later growth stages are considered key physiological factors determining population productivity and nitrogen use efficiency. Nevertheless, the underlying regulatory mechanisms remain poorly understood.

Considerable research has examined the effects of water–fertilizer management or planting density on maize production, yet most studies have treated these factors separately. Although some studies have addressed water–fertilizer or fertilizer–density interactions [21,22], few have integrated water–fertilizer management, planting density, and cultivar genotype within a single experimental framework to systematically evaluate their combined effects. Under high-density cultivation, little is known about how different water–fertilizer regimes affect soil nitrogen distribution, plant nitrogen accumulation, translocation, and allocation, and how these processes influence the relationship between yield formation and water–nitrogen use efficiency. Furthermore, most research on nitrogen use efficiency has focused on nitrogen uptake, with relatively little attention given to within-plant nitrogen translocation and redistribution in high-density populations. To address these gaps, this study selected two widely grown maize cultivars with contrasting tolerance to high planting densities (DH605 and MY73). A split-plot design was implemented, with two water–fertilizer systems—conventional border irrigation (BI) and drip fertigation (DI)—as the main plots and eight planting densities as the subplots. The specific objectives were to: (1) determine the interactive effects of water–fertilizer management and planting density on summer maize yield formation, soil nitrogen distribution, and plant nitrogen accumulation and translocation; (2) assess whether DI improves nitrogen use efficiency in high-density populations by optimizing root-zone nitrogen supply and enhancing nitrogen translocation; and (3) compare varietal responses to water–fertilizer management and planting density.

## 2. Results and Analysis

### 2.1. Effects of Water and Fertilizer Management Methods and Planting Density on Summer Maize Yield and Its Components

A significant interaction between water–fertilizer management and planting density was observed for summer maize yield and its components, and the response patterns differed between cultivars (Table 1 and Table S1). Analysis of variance (ANOVA) revealed significant main effects of water–fertilizer management (WF), variety (V), and planting density (D) on grain yield, as well as significant two-way and three-way interactions among these factors (Table S1), indicating that grain yield formation was co-regulated by irrigation method, varietal characteristics, and planting density. Compared with BI, DI increased grain yield by 7.3% in DH605 and 3.8% in MY73. As planting density increased, ear number per unit area increased continuously in both cultivars, whereas grain number per ear and thousand-grain weight decreased progressively. Consequently, grain yield exhibited a quadratic response to planting density, increasing initially and then declining at higher densities. For DH605, the maximum yield was obtained at D5 under BI and at D6 under DI, while yields at the remaining densities were 3.8–49.3% and 5.3–52.3% lower than the corresponding maxima, respectively. For MY73, the highest yield under both BI and DI was recorded at D7, and yields at the other densities were 6.3–60.4% and 4.7–61.4% lower than the corresponding maxima, respectively. The optimum planting density of MY73 (105,000 plants ha^−1^) was higher than that of DH605 (75,000 plants ha^−1^). In terms of yield components, DH605 was characterized by a greater thousand-grain weight, whereas MY73 produced more ears per unit area and maintained a relatively higher grain number per ear.

### 2.2. Effects of Water and Fertilizer Management Methods and Planting Density on Soil Nitrogen Content

#### 2.2.1. Effects of Water and Fertilizer Management Methods and Planting Density on Soil Total Nitrogen Content

**Figure 1:**
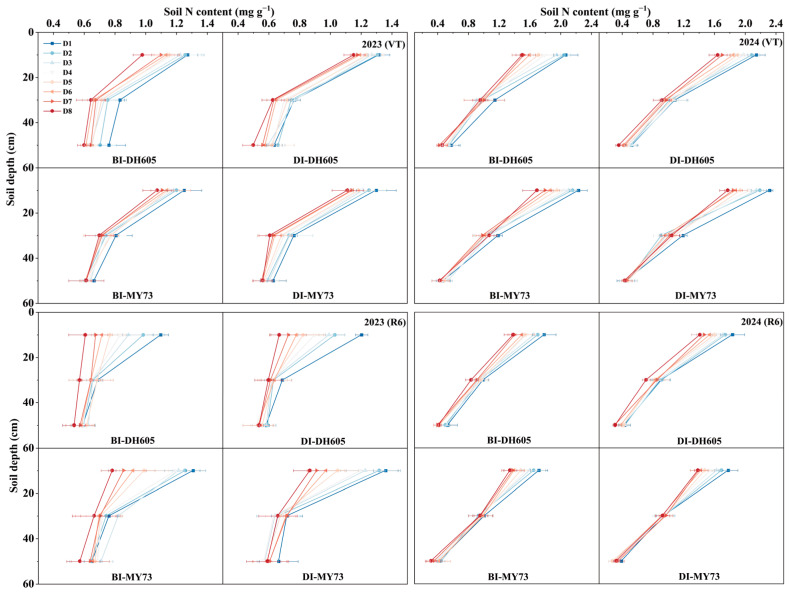
Effects of different water and fertilizer management methods and planting density on soil nitrogen content of summer maize.

Water–fertilizer management and planting density each significantly affected total soil nitrogen content in summer maize, and varietal differences in response were evident (Figure 1). In general, total soil nitrogen content at the VT stage exceeded that at R6, and it declined progressively with depth. Relative to BI, DI primarily elevated total nitrogen content in the topsoil (0–20 cm), whereas no significant differences were found in the 20–60 cm layer. For instance, at the VT stage in 2023, topsoil total nitrogen content under DI was approximately 6.6% and 3.1% higher for DH605 and MY73, respectively, than under BI.

With increasing planting density, total nitrogen content trended downward across all soil layers, with the steepest decline occurring in the 0–20 cm layer, suggesting that high-density stands depleted surface soil nitrogen more intensively. Thus, water–fertilizer management exerted a stronger regulatory effect on the distribution of total nitrogen in the surface soil, whereas planting density affected all layers, although its influence weakened with depth.

#### 2.2.2. Effects of Water and Fertilizer Management Methods and Planting Density on Soil NO_3_^−^-N Content

**Figure 2:**
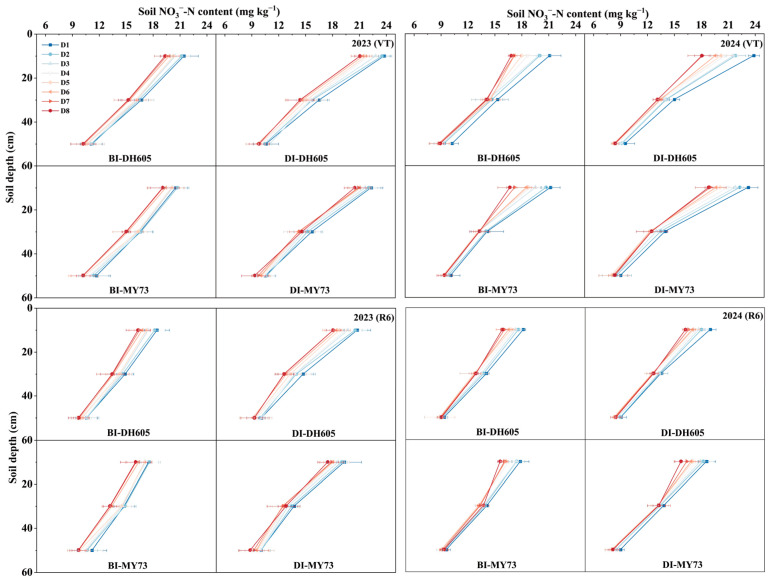
Effects of different water and fertilizer management methods and planting density on soil nitrate nitrogen content of summer maize.

Water–fertilizer management and planting density each significantly influenced soil nitrate-N content, and clear varietal differences were evident (Figure 2). In general, soil nitrate-N content at the VT stage exceeded that at R6 and declined with depth. Relative to BI, DI substantially raised nitrate-N content in the 0–20 cm layer, whereas it was lower in the 20–60 cm layer than under BI, suggesting that DI enhanced nitrate-N accumulation in the topsoil root zone and reduced its residual amounts in deeper layers. For instance, at the VT stage in 2023, nitrate-N content in the 0–20 cm layer under DI was approximately 9.4% and 7.4% higher for DH605 and MY73, respectively, than under BI.

With increasing planting density, nitrate-N content trended downward across all soil layers, with the steepest decline occurring in the topsoil; this decline was more pronounced at VT than at R6. Thus, water–fertilizer management exerted the strongest regulatory effect on the spatial distribution of nitrate-N in the surface soil, whereas planting density affected all layers, although its influence gradually weakened with depth.

#### 2.2.3. Effects of Water and Fertilizer Management Methods and Planting Density on Soil NH4^+^-N Content

**Figure 3:**
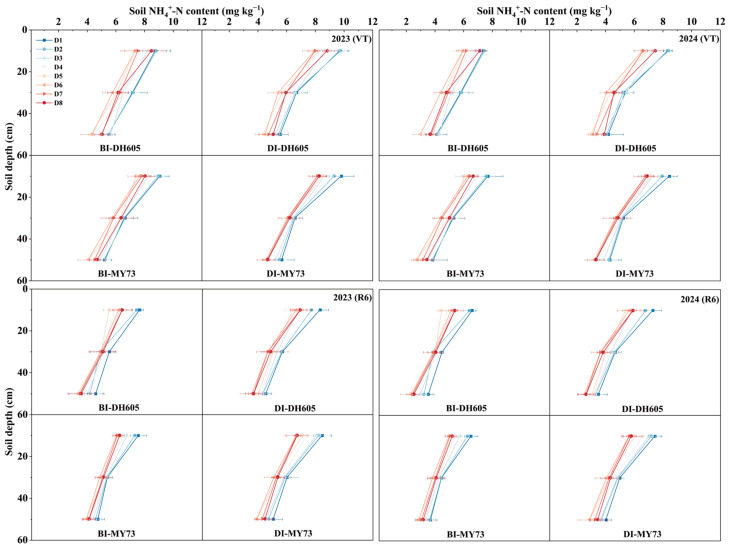
Effects of different water and fertilizer management methods and planting density on soil ammonium nitrogen content of summer maize.

Water–fertilizer management and planting density each significantly influenced soil ammonium-N content, and varietal differences were evident (Figure 3). In general, soil ammonium-N content was higher at VT than at R6 and declined with depth. Relative to BI, DI substantially raised ammonium-N content in the 0–20 cm layer, whereas no significant difference was detected in the 20–60 cm layer. For instance, at the VT stage in 2023, ammonium-N content in the 0–20 cm layer under DI was approximately 8.4% and 7.5% higher for DH605 and MY73, respectively, than under BI.

With increasing planting density, ammonium-N content in the 0–20 cm layer generally trended downward, while in the 20–60 cm layer it remained low and relatively stable. Thus, the effects of water–fertilizer management and planting density on soil ammonium-N were largely confined to the surface soil.

### 2.3. Effects of Water and Fertilizer Management Methods and Planting Density on Nitrogen Accumulation, Translocation, and Utilization Characteristics in Summer Maize

#### 2.3.1. Effects of Water and Fertilizer Management Methods and Planting Density on Nitrogen Accumulation in Summer Maize Plants

**Figure 4:**
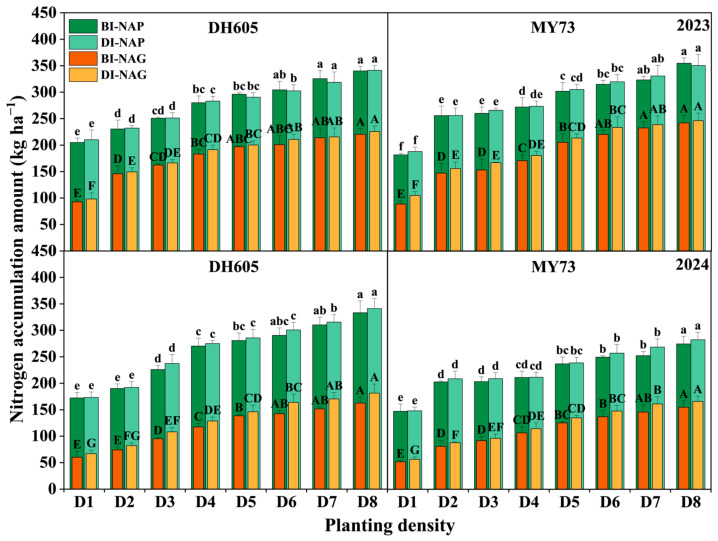
Effects of different water and fertilizer management methods and planting density on nitrogen accumulation in summer maize. Note: Different capital letters indicate significant differences in grain nitrogen accumulation among treatments (*p* < 0.05); different lowercase letters indicate significant differences in plant nitrogen accumulation among treatments (*p* < 0.05). NAP: nitrogen accumulation in plants; NAG: nitrogen accumulation in grains.

Water–fertilizer management and planting density each significantly influenced nitrogen accumulation in maize plants at maturity and in grains, and varietal differences were evident (Figure 4). In general, nitrogen accumulation in both plant tissues and grains of the two varieties rose with increasing planting density, but the increment gradually diminished under high-density treatments (D7–D8). Relative to BI, DI induced only a modest increase in plant nitrogen accumulation, whereas the increase in grain nitrogen accumulation was more substantial. Under DI, nitrogen accumulation in DH605 plants and grains rose by 1.0% and 6.2%, respectively, and in MY73 by 1.8% and 6.4%, indicating that DI favored grain nitrogen accumulation over total plant nitrogen accumulation. Varietal differences in nitrogen accumulation under high-density conditions were also apparent. DH605 exhibited higher plant nitrogen accumulation under some high-density treatments but a relatively weak grain nitrogen advantage; by contrast, MY73 achieved higher grain nitrogen accumulation across most density treatments.

#### 2.3.2. Effects of Water and Fertilizer Management Methods and Planting Density on Nitrogen Translocation in Summer Maize

**Figure 5:**
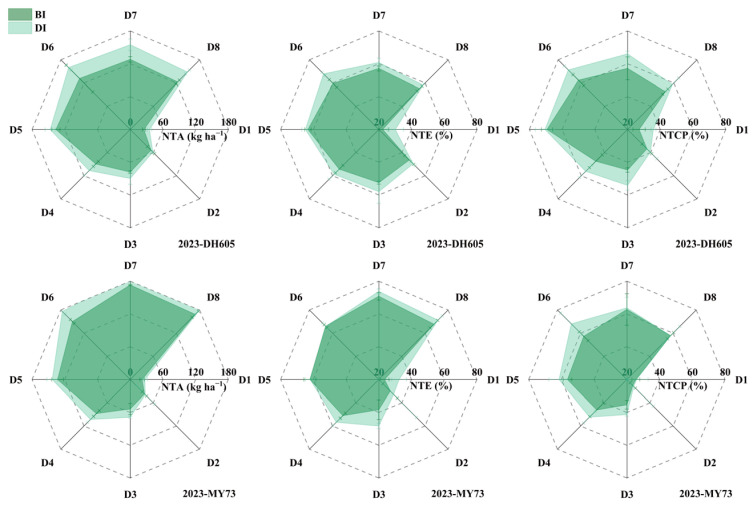
Effects of different water and fertilizer management methods and planting density on nitrogen translocation in summer maize in 2023. Note: Detailed ANOVA results for nitrogen translocation amount (NTA), nitrogen translocation efficiency (NTE), and nitrogen translocation contribution proportion (NTCP) are presented in Table S2.

Water–fertilizer management and planting density each significantly influenced the nitrogen translocation amount (NTA), translocation efficiency (NTE), and translocation contribution proportion (NTCP) of vegetative organs (Figure 5 and Figure S1), and significant interactions were detected (Table S2). ANOVA indicated that water–fertilizer management, variety, and planting density each exerted significant main effects on NTA, NTE, and NTCP, with significant WF × D, V × D, and Y × V × D interactions for NTA (Table S2), suggesting that nitrogen translocation was co-regulated by irrigation method, varietal characteristics, and planting density. Overall, relative to BI, DI substantially enhanced all three nitrogen translocation parameters in both varieties: for DH605, NTA rose by 18.5%, NTE by 12.5%, and NTCP by 9.5%; for MY73, the corresponding increases were 17.8%, 13.0%, and 13.9%, respectively.

As planting density increased, the NTA, NTE, and NTCP of both varieties showed a trend of first rising and then falling. For DH605, the highest NTA under BI and DI treatments occurred at the D5 and D6 treatments, respectively, with NTA in the other treatments decreasing by 6.8–78.5% and 9.9–75.0% compared with the peak treatments. For MY73, the highest NTA under both BI and DI treatments occurred in the D7 treatment, with NTA in the other treatments decreasing by 7.7–83.9% and 5.0–81.1% compared with the peak treatments. Differences in nitrogen translocation capacity between the two varieties at high densities were observed: under D6–D8 treatments, NTA of MY73 was generally higher than that of DH605.

#### 2.3.3. Effects of Water and Fertilizer Management Methods and Planting Density on Nitrogen Utilization Characteristics of Summer Maize

**Figure 6:**
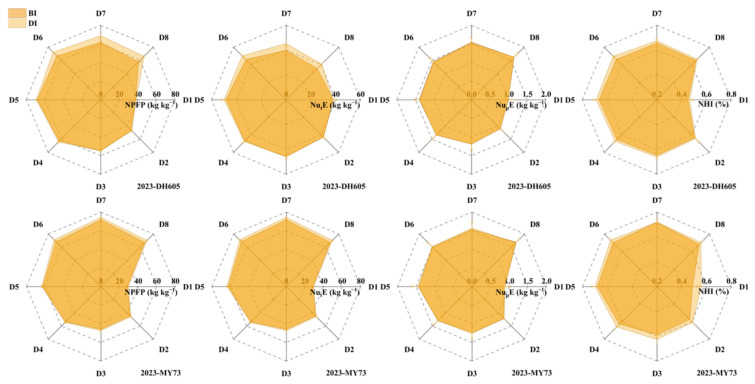
Effects of different water and fertilizer management methods and planting density on nitrogen partial factor productivity, nitrogen utilization efficiency, nitrogen uptake efficiency, and nitrogen harvest index in summer maize in 2023. Note: Detailed ANOVA results for nitrogen partial factor productivity (NPFP), nitrogen utilization efficiency (NU_t_E), nitrogen uptake efficiency (NU_p_E), and nitrogen harvest index (NHI) are presented in Table S3.

Water–fertilizer management and planting density exerted significant effects on nitrogen partial factor productivity (NPFP), nitrogen utilization efficiency (NU_t_E), and nitrogen harvest index (NHI) of summer maize, with interactive effects; however, their influence on nitrogen uptake efficiency (NU_p_E) was negligible (Figure 6 and Figure S2, Table S3). Compared with BI, DI significantly increased NPFP, NU_t_E, and NHI in both cultivars, with DH605 increasing by 7.4%, 5.4%, and 5.4%, respectively, and MY73 increasing by 3.5%, 1.4%, and 18.0%, respectively. As planting density increased, NPFP, NU_t_E, NHI, and NU_p_E all showed a trend of first rising and then declining. Taking NU_t_E as an example, under BI treatment, DH605 exhibited the highest NU_t_E at D5, with other treatments decreasing by 2.2–29.2% compared to the peak treatment; under DI treatment, the peak occurred at D6, with other treatments decreasing by 0.8–22.1%. For MY73, the highest NU_t_E under both BI and DI treatments occurred at D7, with other treatments decreasing by 1.3–31.3% compared with the peak. Comparing the two cultivars, MY73 showed generally higher NPFP and NU_t_E than DH605 under high-density treatments D6–D8, indicating stronger nitrogen use capability under high-density populations. Notably, DI significantly improved NU_t_E and NHI, while its effect on NU_p_E was not significant.

### 2.4. Effects of Water and Fertilizer Management Methods and Planting Density on Water Use Efficiency in Summer Maize

**Figure 7:**
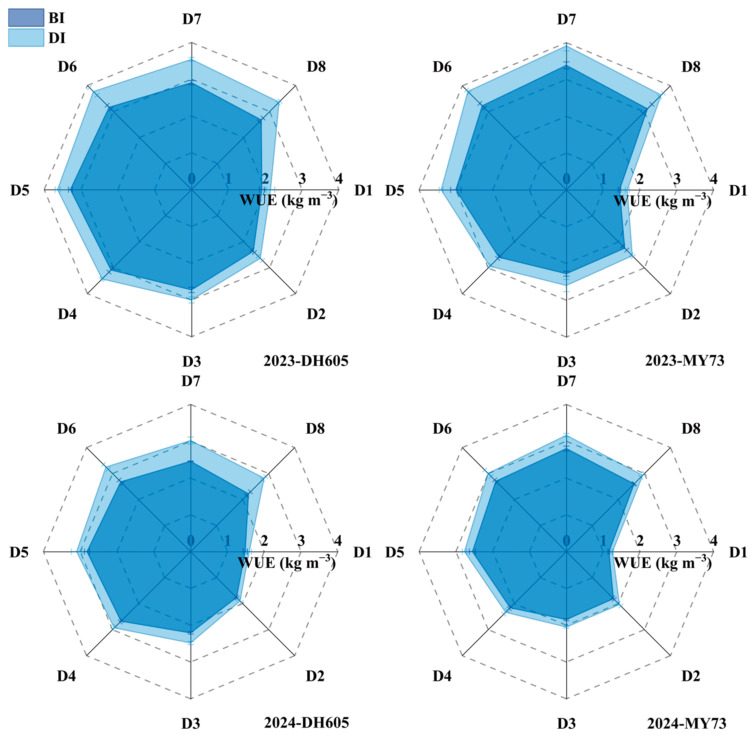
Effects of different water and fertilizer management methods and planting density on water use efficiency of summer maize in 2023 and 2024. Note: Detailed ANOVA results for water use efficiency (WUE) are presented in Table S3.

Water–fertilizer management and planting density each significantly influenced summer maize water use efficiency (WUE), and their interaction was significant (Figure 7, Table S3). ANOVA results showed that water–fertilizer management, variety, and planting density each exerted significant main effects on WUE, and the WF × D, WF × V, and WF × V × D interactions were also significant (Table S3), indicating that WUE was co-regulated by irrigation method, varietal characteristics, and planting density. Overall, compared with BI, DI significantly increased WUE for both varieties, with DH605 and MY73 increasing by 18.4% and 16.3%, respectively. As planting density increased, WUE for both varieties showed a trend of first increasing and then decreasing. For DH605 under BI treatment, the highest WUE occurred at the D5 treatment, with the other treatments decreasing by 4.7–46.6% compared with the peak; under DI treatment, the peak occurred at D6, with the other treatments decreasing by 4.1–49.0%. For MY73, the highest WUE under both BI and DI treatments occurred at the D7 treatment, with the other treatments decreasing by 3.5–58.4% compared with the peak. DH605 was more sensitive to changes in density, with the WUE peak occurring at a lower density than MY73; under DI treatment, MY73’s WUE peak could be maintained up to the D7 treatment, and the decline at D8 was smaller than that of DH605.

## 3. Discussion

### 3.1. Effects of Water and Fertilizer Management Methods and Planting Density on Summer Maize Yield and Its Components

Achieving high crop yields depends on the coordination between population structure and resource supply, and water and fertilizer management methods, as well as planting density, are key factors influencing summer maize yield formation [23,24]. The results of this study indicated that drip irrigation combined with integrated water–fertilizer management (DI), together with optimal planting density, could significantly increase summer maize yield, and different varieties exhibited distinct responses to planting density as well as water–fertilizer management methods (Table 1 and Table S1). Previous studies have shown that there is a certain compensatory relationship among ear number per unit area, grain number per ear, and thousand-grain weight, and their responses to changes in planting density are not synchronized [25,26]. Moderate density increase can boost yield by raising ear number per population, but excessively high density intensifies competition for light, water, and nutrients among plants, thus limiting grain formation and leading to yield reduction [14]. Under the experimental conditions of this study, DH605 achieved higher yield levels at 75,000–90,000 plants ha^−1^, whereas MY73 could still maintain high yields at 105,000 plants ha^−1^, indicating significant differences in density tolerance among varieties.

Compared with BI treatment, DI treatment maintained relatively higher grain number per ear and thousand-grain weight under higher density conditions, indicating that optimizing water and fertilizer supply can alleviate resource competition pressure under high-density populations and is beneficial for population yield formation. Previous studies have shown that precise and split applications of water and fertilizer help improve the stability of water and nitrogen supply in the root zone during critical crop growth stages and promote the coordination between water and nitrogen demand [27,28], thereby enhancing grain formation capacity during the grain-filling period and supporting higher population yields [29,30]. Combined with the subsequent nitrogen accumulation and translocation results, it could be seen that the increased yield of high-density populations under DI treatment depended not only on the improved water and nitrogen supply in the root zone but was also closely related to the enhanced translocation of nitrogen from vegetative tissues to grains during the later growth stages. In addition, different varieties showed significant differences in response to DI treatment. MY73 had already exhibited strong density tolerance under BI treatment, so the yield increase brought by DI treatment was relatively limited; in contrast, DH605 showed more pronounced improvement in high-density adaptability under DI treatment, indicating that varieties with weaker density tolerance rely more on optimized water and fertilizer conditions to promote high-density population yield formation. Especially under high-density conditions, DI can mitigate the inhibitory effects of resource supply-demand imbalance on individual growth and grain formation, thereby enhancing population stability and unleashing yield potential [31,32,33]. Therefore, in high-yield and high-efficiency summer maize cultivation, water and fertilizer management methods should be reasonably matched with planting density according to the density tolerance characteristics of the variety to achieve synergistic improvement of yield and resource use efficiency.

### 3.2. Effects of Water and Fertilizer Management Methods and Planting Density on Nitrogen Utilization Characteristics of Summer Maize

The absorption, accumulation, translocation, and distribution of nitrogen directly affect crop dry matter formation and grain yield [34,35]. The results of this study indicated that DI treatment significantly increased the nitrogen accumulation in mature plants and grains of DH605 and MY73 (Figure 4). Compared with BI, DI showed significant differences in nitrogen content across soil layers, indicating that DI promoted nitrogen enrichment in the root zone and optimized nitrogen spatial distribution (Figure 1, Figure 2 and Figure 3). The increase in ammonium nitrogen content might be due to split fertilization reducing nitrogen losses through ammonia volatilization and denitrification, which helped maintain higher levels of available nitrogen in the root zone, thereby enhancing effective nitrogen supply in the root area. Under BI treatment, the higher nitrate nitrogen content in the 20–60 cm soil layer might be related to nitrogen leaching under border irrigation conditions [22,36,37]. In contrast, DI treatment, through a split fertilization strategy, facilitated the temporal and spatial matching of nitrogen supply with crop demand, thereby promoting the accumulation of both dry matter and nitrogen [11,12,38].

There were significant interactive effects of water and fertilizer management methods and planting density on nitrogen accumulation and translocation (Tables S2 and S3). As planting density increased, the population’s nitrogen demand rose, but excessive density suppressed individual growth, resulting in a decreased capacity for vegetative organs to translocate nitrogen to the grains [39]. The DI treatment significantly increased the nitrogen translocation amount, translocation efficiency, and translocation contribution rate compared with the BI treatment, and maintained relatively high grain yield under higher density conditions. Split fertilization during key growth stages in the DI treatment helped promote the continuous translocation of nitrogen from vegetative organs to the grains, thereby alleviating the decline in grain number per ear and thousand-grain weight under high-density conditions [10,40]. Notably, the improvement of nitrogen use efficiency under the DI treatment did not rely on an increase in total nitrogen uptake. In this study, the DI treatment had no significant impact on NU_p_E, but NTA, NTE, and NTCP were all significantly improved (Figure 5, Figure 6, Figures S1 and S2, Tables S2 and S3), indicating that DI primarily enhanced nitrogen use efficiency by promoting nitrogen redistribution rather than increasing nitrogen uptake. One possible explanation for the limited effect of drip fertigation on NU_p_E is that the total nitrogen application rate was identical between DI and BI treatments, resulting in a similar overall nitrogen supply and hence limited potential for increasing total nitrogen uptake. In contrast, drip fertigation promoted the remobilization and translocation of nitrogen from vegetative organs to developing grains rather than substantially increasing total nitrogen acquisition. This finding may represent a key breakthrough for improving nitrogen use efficiency under high-density conditions.

Nitrogen use efficiency is an important indicator for evaluating the sustainability of agricultural production [41,42]. In this study, the DI treatment not only increased yield but also significantly improved NPFP, NU_t_E, and NHI, while maintaining a relatively high level of NU_t_E under high-density conditions (Figure 6 and Figure S2). This indicated that optimized water and fertilizer management was conducive to maintaining nitrogen use efficiency under high population density conditions and promoted coordination between grain formation and dry matter accumulation. Previous studies have shown that reasonable water–nitrogen co-management can enhance crop nitrogen absorption and utilization [34,43], and a moderate increase in planting density also helps improve population resource utilization capacity [44,45]. There were significant differences in nitrogen use efficiency between the two varieties (Figure 5, Figure 6, Figures S1 and S2), which were consistent with their differences in density tolerance. The more density-tolerant variety (MY73) exhibited higher NTA and NU_t_E, whereas the less density-tolerant variety (DH605) showed a greater increase in nitrogen use efficiency under drip fertigation. Therefore, varieties with weaker density tolerance rely more on optimized water and fertilizer management to enhance nitrogen redistribution into grains. The physiological mechanisms underlying these varietal differences provide an important basis for developing differentiated water–nitrogen management strategies according to varietal density tolerance in the future. This study further demonstrated that integrating drip irrigation with fertilization and reasonable planting density was conducive to the combined improvement of summer maize yield and nitrogen use efficiency.

### 3.3. Effects of Water and Fertilizer Management Methods and Planting Density on Water Use Efficiency in Summer Maize

Water use efficiency (WUE) is an important indicator for assessing the relationship between crop production and water resource consumption [28]. The results of this study indicated that optimizing water and fertilizer management can maintain high yield per unit of water consumption under high-density planting conditions. Moderate planting density was conducive to improving the population’s ability to utilize resources such as light and water, thereby enhancing the efficiency of yield formation per unit of water consumed. However, when the density exceeded a certain range, water competition within the population intensified, resource consumption increased, and WUE declined. In this study, the WUE of both varieties first increased and then decreased with increasing planting density, while under the DI treatment, the peak WUE occurred at a higher density level (Figure 7), indicating that optimizing water and fertilizer supply helped maintain population water use efficiency under high-density conditions [6,46]. From the perspective of WUE composition, there were two complementary pathways through which DI treatment enhanced WUE: on the water consumption side, precise split water supply reduced deep percolation and ineffective evaporation [47,48,49]; on the yield formation side, synchronizing water and fertilizer supply enhanced the water–nitrogen synergistic effect during critical growth periods, which was beneficial for maintaining a high rate of dry matter accumulation during the grain-filling stage [50,51]. The combined effect of these two pathways allowed DI treatment to support higher population density without significantly increasing water consumption per unit area, thereby achieving a synergistic increase in WUE. Overall, drip irrigation with integrated water and fertilizer management significantly improved WUE while maintaining high yields and was an effective approach to promoting the coordinated improvement of yield and water use efficiency [52,53].

### 3.4. Limitations and Future Perspectives

Although this study elucidated the regulatory roles of drip fertigation in optimizing root-zone nitrogen distribution and enhancing plant nitrogen translocation under high-density conditions, several limitations should be acknowledged. First, the present study mainly focused on nitrogen accumulation and redistribution processes, whereas the physiological coordination between carbon assimilation and nitrogen utilization was not directly examined. Because nitrogen metabolism is closely coupled with photosynthetic carbon fixation and source–sink dynamics, future studies should integrate measurements of photosynthetic performance, carbon assimilation capacity, and key nitrogen metabolism enzymes to further clarify the physiological mechanisms underlying yield improvement and enhanced nitrogen use efficiency under drip fertigation. Second, this study was conducted over two growing seasons, and the long-term impacts of drip fertigation on soil nitrogen cycling, microbial community composition, soil health, and greenhouse gas emissions under high-density planting conditions remain unclear. Although drip fertigation promoted nitrogen enrichment in the root zone and reduced nitrate accumulation in deeper soil layers during the experimental period, the long-term environmental consequences of continuous drip fertigation management require further evaluation. Potential environmental risks, including nitrate leaching, groundwater contamination, alterations in soil microbial communities, and changes in greenhouse gas emissions, may emerge under prolonged intensive production conditions and cannot be fully assessed through short-term experiments. Therefore, long-term field studies are needed to comprehensively evaluate the sustainability, environmental risks, and ecological consequences of intensive drip fertigation systems. In addition, although a detailed economic evaluation was not conducted in this study, the observed improvements in grain yield, water use efficiency, and nitrogen utilization efficiency suggest that drip fertigation may improve input–output efficiency compared with conventional flood irrigation, indicating potential economic advantages in high-density maize production systems. Finally, significant varietal differences in density tolerance and nitrogen utilization characteristics were observed in this study. These findings suggest that future research should integrate cultivar improvement with precision water–nitrogen management strategies to optimize the synergistic regulation of yield formation and resource use efficiency in high-density maize production systems.

## 4. Materials and Methods

### 4.1. Overview of the Experimental Site

This experiment was conducted from 2023 to 2024 in Dawenkou Town, Tai’an City, Shandong Province (35°97′ N, 117°01′ E, elevation 178 m). The study site features a typical temperate continental monsoon climate, with an annual average precipitation of 735.6 mm, a frost-free period of approximately 195 days, and an annual average temperature of 13.9 °C. The soil type in this region is brown loam soil, with an average bulk density of 1.6 g cm^−3^ in the 0–100 cm soil layer, an average field capacity of 23%, and an average wilting coefficient of 10%.

### 4.2. Experimental Design

The summer maize varieties Denghai 605 (DH605) and MY73 were selected as test materials. A split-plot design was employed, with water and fertilizer management serving as the main plots and planting density as the subplots. The two water and fertilizer management treatments included conventional border irrigation (BI) and drip fertigation (DI). In BI, a compound fertilizer was applied as a single basal application at sowing (N:P_2_O_5_:K_2_O = 28:7:9), followed by one irrigation event after sowing, with a total irrigation amount of 100 mm in 2023 and 50 mm in 2024. In DI, phosphorus and potassium fertilizers were applied as a basal application before sowing, while nitrogen fertilizer was applied through fertigation in four split applications at the sowing, jointing, large whorl, and 15 days after the tasseling stage, with a ratio of 1:2:5:2. Irrigation was also applied in four events, with total amounts of 60 mm in 2023 and 30 mm in 2024. Irrigation scheduling in this study was determined based on local high-yield maize production practices in the North China Plain, combined with long-term seasonal precipitation patterns and crop water demand characteristics. The irrigation strategy was designed to ensure that soil water conditions remained close to those commonly adopted in regional high-yield management systems, while avoiding water stress during critical growth stages. In addition, the irrigation amounts were adjusted according to annual precipitation distribution to better reflect practical field conditions and to ensure comparability with previous regional studies on maize water management under both border irrigation and drip fertigation systems. The fertilization amounts for the two water and fertilizer management methods were the same, namely N at 210 kg ha^−1^ (UAN, urea ammonium nitrate, was applied in the DI treatment), K_2_O at 67.5 kg ha^−1^, and P_2_O_5_ at 52.5 kg ha^−1^. The eight planting density treatments were 15,000 (D1), 30,000 (D2), 45,000 (D3), 60,000 (D4), 75,000 (D5), 90,000 (D6), 105,000 (D7), and 120,000 plants ha^−1^ (D8). There were 32 treatments in total (Table 2), each treatment had 3 replicates, and the area of each plot was 180 m^2^. Irrigation pipes were laid after sowing; in the DI treatment, the spacing between the drip tape and the maize rows was 0.1 m. The sowing dates were 21 June 2023 and 21 June 2024, and the harvest dates were 5 October 2023 and 30 September 2024. Field management was carried out according to high-yield field practices.

### 4.3. Measurement Items and Methods

#### 4.3.1. Soil Nitrogen Content

Soil samples were collected before sowing (S), at the tasseling stage (VT), and at maturity (R6). Soil was cored in 20 cm layers from the 0–60 cm depth range, with three replicates per treatment, and stored at low temperatures. Fresh soil samples were extracted using 1 mol L^−1^ KCl, and the extract was analyzed for nitrate nitrogen and ammonium nitrogen content using a continuous flow analyzer (AA3, Norderstedt, Germany). After air-drying and grinding, soil samples were digested with H_2_SO_4_-H_2_O_2_, and the digest was analyzed for total nitrogen content using a continuous flow analyzer (AA3, Germany).

#### 4.3.2. Crop Evapotranspiration (ET_c_) and Water Use Efficiency (WUE)

Crop evapotranspiration was calculated using the soil water balance equation:

ETc = P + I + C + (SWS1-SWS2) − D − R

In the equation, ET_c_ represented crop evapotranspiration during the maize growing season (mm); P (mm) represented precipitation during the summer maize growing season; I (mm) represented irrigation volume; C (mm) represented groundwater recharge; since the groundwater table was approximately 80 m below the soil surface, this term was neglected; SWS_1_ (mm) and SWS_2_ (mm) represented soil water storage in the 0–100 cm soil layer at pre-sowing and maturity, respectively; R (mm) represented surface runoff; since the terrain in all plots was flat and no runoff occurred during the experiment, this term was neglected. D (mm) represented deep percolation. Since there were multiple heavy rainstorms during the summer maize growing season, D had to be considered. It was calculated as the total available water content (mm) in the 0–100 cm soil layer before irrigation + irrigation volume (mm) − field capacity (mm).

Water use efficiency (WUE) was defined as the ratio of grain yield (t ha^−1^) to ET_c_ (mm), calculated as follows:

WUE = GY/ETc

In the equation, GY represented crop yield (t ha^−1^); ET_c_ represented crop evapotranspiration (mm).

#### 4.3.3. Nitrogen Accumulation and Translocation in Plants

The dried plant samples were ground separately by organ, digested with H_2_SO_4_-H_2_O_2_, and the total nitrogen content of each plant organ was measured using a flow analyzer (AA3, Germany). The formulas for calculating nitrogen accumulation and translocation in maize plants are as follows:

Nitrogen accumulation in each organ = nitrogen content in each organ × dry matter weight of each organ

Nitrogen translocation amount in vegetative organs (NTA, kg ha^−1^) = nitrogen accumulation in vegetative organs at the silking stage − nitrogen accumulation in vegetative organs at maturity

Nitrogen translocation efficiency (NTE, %) = (nitrogen translocation amount in vegetative organs/nitrogen accumulation in vegetative organs at the silking stage) × 100

Nitrogen translocation contribution percentage (NTCP, %) = (nitrogen translocation amount in vegetative organs/nitrogen accumulation in grain at maturity) × 100.

#### 4.3.4. Nitrogen Use Efficiency

Based on grain yield measured at physiological maturity, nitrogen accumulation in various plant organs, and nitrogen application rates, nitrogen use efficiency was calculated as follows:

Nitrogen utilization efficiency (NU_t_E, kg kg^−1^) = grain yield/plant nitrogen accumulation

Nitrogen uptake efficiency (NU_p_E, kg kg^−1^) = nitrogen accumulation in mature plants/nitrogen applied

Nitrogen partial factor productivity (NPFP, kg kg^−1^) = grain yield/nitrogen application rate

Nitrogen harvest index (NHI, %) = (nitrogen accumulation in grains at maturity/nitrogen accumulation in the plant at maturity) × 100%.

#### 4.3.5. Yield and Its Components

At the maturity stage (R6), 30 ears were randomly selected from the middle six rows of each plot for manual harvesting, with three replicates. The number of plants per unit area, ear number, and the number of grains per ear were determined. After threshing, 1000 seeds were counted manually with nine replicates and dried at 60 °C to constant weight to analyze crop yield (with a moisture content of 14%).

Grain yield (t ha^−1^) = ear number per hectare × grain number per ear × 1000-grain weight.

#### 4.3.6. Statistical Analysis

Analysis of variance (ANOVA) was performed using IBM SPSS Statistics 27.0 to evaluate the effects of year (Y), water and fertilizer management (WF), variety (V), planting density (D), and their interactions on measured variables. When significant effects were detected, the least significant difference (LSD) test was used for multiple comparisons at *p* < 0.05. Microsoft Excel 2019 was used for data processing, and figures were plotted using Origin 2021. Detailed ANOVA results are provided in Tables S1–S3.

## 5. Conclusions

Drip fertigation (DI) significantly improved grain yield, water use efficiency (WUE), and nitrogen utilization efficiency (NU_t_E) in high-density summer maize. Grain yield increased by 7.3% and 3.8%, WUE by 18.4% and 16.3%, and NPFP by 7.4% and 3.5% for DH605 and MY73, respectively. Planting density significantly influenced crop performance, with optimal density being cultivar-dependent (105,000 plants ha^−1^ for MY73 and 75,000–90,000 plants ha^−1^ for DH605), indicating the importance of matching density with varietal characteristics under drip fertigation. DI enhanced root-zone nitrogen availability and post-silking nitrogen translocation to grains, improving NU_t_E without affecting NU_p_E. Overall, DI promotes coordinated improvement of yield, WUE, and NU_t_E by optimizing root-zone nitrogen distribution and enhancing internal nitrogen remobilization in high-density summer maize.

## Figures and Tables

**Table 1:** Effects of different water and fertilizer management methods and planting density on yield and its components of summer maize.

Year	Water and Fertilizer Management Method	Variety	Planting Density	Number of Ears (No. ha^−1^)	Grains per Ear	1000-Grain Weight (g)	Grain Yield (t ha^−1^)
2023	BI	DH605	D1	32,986 m	588.9 h	403.1 ab	7.8 p
			D2	36,458 kl	661.9 a	406.4 a	9.8 n
			D3	46,528 j	635.4 b	387.6 d	11.5 m
			D4	57,292 gh	605.8 c	381.1 e	13.2 jk
			D5	69,444 f	563.5 lm	366.2 f	14.3 efg
			D6	81,944 e	491.4 r	346.4 g	13.9 gh
			D7	89,583 d	427.5 u	338.5 h	13.0 k
			D8	94,097 b	397.1 v	323.4 lm	12.1 l
		MY73	D1	34,722 lm	562.1 m	306.5 no	6.0 q
			D2	55,556 h	527.1 o	319.8 m	9.4 o
			D3	48,611 i	598.3 ef	331.4 ij	9.6 no
			D4	57,986 g	593.4 g	326.8 jkl	11.2 m
			D5	69,792 f	585.7 i	321.4 lm	13.1 jk
			D6	81,597 e	574.3 k	305.7 no	14.3 efg
			D7	89,583 d	562.9 lm	298.9 p	15.1 bc
			D8	98,611 a	506.4 q	281.9 q	14.1 fgh
	DI	DH605	D1	33,333 m	601.3 d	398.6 bc	8.0 p
			D2	37,153 k	664.7 a	401.0 ab	9.9 n
			D3	46,181 j	636.3 b	394.1 c	11.6 m
			D4	58,333 g	606.0 c	381.3 e	13.5 ij
			D5	69,792 f	563.8 lm	369.3 f	14.5 de
			D6	82,639 e	531.5 n	345.6 g	15.2 b
			D7	93,403 bc	466.2 s	332.3 ij	14.5 ef
			D8	99,653 a	432.0 t	322.0 lm	13.9 hi
		MY73	D1	35,069 lm	565.5 l	311.6 n	6.2 q
			D2	56,250 gh	532.8 n	320.8 lm	9.6 no
			D3	49,306 i	600.8 de	335.3 hi	9.9 n
			D4	58,333 g	595.5 fg	330.2 ijk	11.5 m
			D5	70,139 f	587.4 hi	324.4 klm	13.4 jk
			D6	82,639 e	582.5 j	310.5 n	14.9 bcd
			D7	91,667 c	564.8 lm	302.1 op	15.6 a
			D8	99,306 a	524.3 p	283.1 q	14.7 cde
2024	BI	DH605	D1	30,833 n	560.5 ijk	394.3 a	6.8 p
			D2	31,667 n	651.4 a	395.5 a	8.2 o
			D3	45,278 l	599.3 c	381.7 b	10.4 l
			D4	59,444 h	582.8 fg	366.0 c	12.7 h
			D5	71,944 g	545.1 l	342.9 d	13.4 de
			D6	84,167 f	467.9 r	327.1 f	12.9 fgh
			D7	91,667 d	400.5 u	324.5 fg	11.9 j
			D8	92,222 d	368.4 v	319.8 g	10.9 k
		MY73	D1	33,611 m	559.6 jk	284.1 l	5.3 q
			D2	56,111 j	524.4 o	286.8 kl	8.4 no
			D3	48,889 k	594.4 d	297.7 hi	8.7 mn
			D4	58,889 h	590.1 e	293.6 ij	10.2 l
			D5	72,222 g	581.0 g	290.7 jk	12.2 ij
			D6	85,000 ef	571.6 h	268.3 m	13.0 fgh
			D7	95,000 c	545.6 l	261.6 no	13.6 d
			D8	100,000 a	497.7 q	256.3 o	12.8 gh
	DI	DH605	D1	30,833 n	564.1 i	396.0 a	6.9 p
			D2	31,944 n	653.1 a	397.0 a	8.3 o
			D3	45,278 l	616.7 b	391.0 a	10.9 k
			D4	59,444 h	585.2 f	376.5 b	13.1 fg
			D5	72,500 g	559.4 jk	345.5 d	14.0 c
			D6	84,167 f	530.2 n	335.1 e	15.0 a
			D7	96,111 bc	445.7 s	326.0 f	14.0 c
			D8	99,444 a	410.7 t	321.8 fg	13.1 ef
		MY73	D1	33,611 m	561.9 ij	286.0 kl	5.4 q
			D2	57,222 ij	535.8 m	288.0 jkl	8.8 m
			D3	49,167 k	600.1 c	302.9 h	8.9 m
			D4	58,056 hi	593.5 de	298.7 hi	10.3 l
			D5	72,500 g	583.5 fg	291.7 jk	12.3 i
			D6	86,111 e	580.6 g	271.5 m	13.6 d
			D7	96,944 b	556.6 k	266.7 mn	14.4 b
			D8	100,278 a	520.5 p	258.2 o	13.5 de

Note: Different letters within the same column indicate significant differences among treatments at *p* < 0.05. BI, conventional border irrigation; DI, drip fertigation. D1–D8 represent planting densities of 15,000, 30,000, 45,000, 60,000, 75,000, 90,000, 105,000, and 120,000 plants ha^−1^, respectively.

**Table 2:** Experimental factors and treatment levels used in the study.

Factor	Levels
Water–fertilizer management	BI (border irrigation), DI (drip fertigation)
Cultivar	DH605, MY73
Planting density	D1 (15,000), D2 (30,000), D3 (45,000), D4 (60,000), D5 (75,000), D6 (90,000), D7 (105,000), D8 (120,000 plants ha^−1^)
Nitrogen application rate	210 kg ha^−1^
Phosphate Fertilizer application rate	52.5 kg ha^−1^
Potash fertilizer application rate	67.5 kg ha^−1^
Irrigation volume	BI (100 mm in 2023 and 50 mm in 2024) DI (60 mm in 2023 and 30 mm in 2024)
Irrigation frequency	BI (1 time per season (2023, 2024)) DI (4 times per season (2023, 2024))
Experimental design	2 × 2 × 8 factorial design with three replications

## Data Availability

The original contributions presented in this study are included in the article/Supplementary Materials. Further inquiries can be directed to the corresponding authors.

## Supplementary Materials

The following supporting information can be downloaded at: https://www.mdpi.com/article/10.3390/plants15132026/s1, Figure S1: Effects of different water and fertilizer management methods and planting density on nitrogen translocation in summer maize in 2024; Figure S2: Effects of different water and fertilizer management methods and planting density on nitrogen partial factor productivity, nitrogen utilization efficiency, nitrogen uptake efficiency, and nitrogen harvest index in summer maize in 2024; Table S1: Analysis of variance (ANOVA) for yield and its components; Table S2: Analysis of variance (ANOVA) for nitrogen translocation amount (NTA), nitrogen translocation efficiency (NTE), and nitrogen translocation contribution proportion (NTCP) in vegetative organs; Table S3: Analysis of variance (ANOVA) for water use efficiency (WUE), nitrogen partial factor productivity (NPFP), nitrogen utilization efficiency (NU_t_E), nitrogen uptake efficiency (NU_p_E) and nitrogen harvest index (NHI).

## Abbreviations

The following abbreviations are used in this manuscript:

DI	Drip fertigation
BI	Border irrigation
DH605	Denghai 605
ET_c_	Crop evapotranspiration
WUE	Water use efficiency
NTA	Nitrogen translocation amount
NTE	Nitrogen translocation efficiency
NTCP	Nitrogen translocation contribution percentage
NHI	Nitrogen harvest index
NU_p_E	Nitrogen uptake efficiency
NU_t_E	Nitrogen utilization efficiency
NPFP	Nitrogen partial factor productivity
ANOVA	Analysis of variance
LSD	Least significant difference
NO_3_^−^-N	Nitrate nitrogen
NH_4_^+^-N	Ammonium nitrogen
S	Sowing
VT	Tasseling stage
R6	Maturity
