# Supplementary material for: Drip Fertigation Optimizes the Spatial Distribution and Translocation of Nitrogen, Thereby Increasing Yields and Improving Water and Nitrogen Use Efficiency in High-Density Summer Maize
Source: Plants (Basel). 2026 Jun 30;15(13):2026. doi: 10.3390/plants15132026 (PMC13364147; doi:10.3390/plants15132026)
Supplement: Supplementary file 1 [file plants-15-02026-s001.zip › plants-4391167-supplementary.pdf]

Supplementary data :

Table S1 Analysis of variance (ANOVA) for yield and its components.

Source of variation	Number of ears (No. ha^{-1})	Grains per ear	1000-grain weight (g)	Grain yield (t ha^{-1})
Year (Y)	**	**	**	**
Water and fertilizer management method (WF)	**	**	**	**
Variety (V)	**	**	**	**
Planting density (D)	**	**	**	**
Y×WF	ns	**	**	**
Y×V	**	**	**	**
Y×D	**	**	**	**
WF×V	**	**	ns	**
WF×D	**	**	*	**
V×D	**	**	**	**
Y×WF×V	ns	**	**	**
Y×WF×D	ns	**	ns	ns
Y×V×D	**	**	**	**
WF×V×D	**	**	ns	**
Y×WF×V×D	ns	**	ns	ns

Note: ns: not significant; * indicates significant differences at $P < 0.05$, ** indicates significant differences at $P < 0.01$, the same as below.

Table S2 Analysis of variance (ANOVA) for nitrogen translocation amount (NTA), nitrogen translocation efficiency (NTE), and nitrogen translocation contribution proportion (NTCP) in vegetative organs.

Source of variation	NTA	NTE	NTCP
Year (Y)	**	**	ns
Water and fertilizer management method (WF)	**	**	**
Variety (V)	ns	**	**
Planting density (D)	**	**	**
Y×WF	ns	ns	ns
Y×V	ns	ns	ns
Y×D	**	**	**
WF×V	ns	ns	ns
WF×D	*	ns	ns
V×D	**	**	*
Y×WF×V	ns	ns	ns
Y×WF×D	ns	ns	ns
Y×V×D	*	ns	ns
WF×V×D	ns	ns	ns
Y×WF×V×D	ns	ns	ns

Table S3 Analysis of variance (ANOVA) for water use efficiency (WUE), nitrogen partial factor productivity (NPFP), nitrogen use efficiency (NU_tE), nitrogen uptake efficiency (NU_pE) and nitrogen harvest index (NHI).

Source of variation	WUE	NPFP	NU_tE	NU_pE	NHI
Year (Y)	**	**	ns	**	**
Water and fertilizer management method (WF)	**	**	**	ns	**
Variety (V)	**	**	**	ns	**
Planting density (D)	**	**	**	**	**
Y×WF	**	**	ns	ns	ns
Y×V	ns	**	ns	ns	**
Y×D	**	**	ns	ns	**
WF×V	**	**	ns	ns	ns
WF×D	**	**	ns	ns	ns
V×D	**	**	**	ns	**
Y×WF×V	ns	**	ns	ns	ns
Y×WF×D	ns	ns	ns	ns	ns
Y×V×D	**	**	ns	ns	**
WF×V×D	*	**	ns	ns	ns
Y×WF×V×D	ns	ns	ns	ns	ns

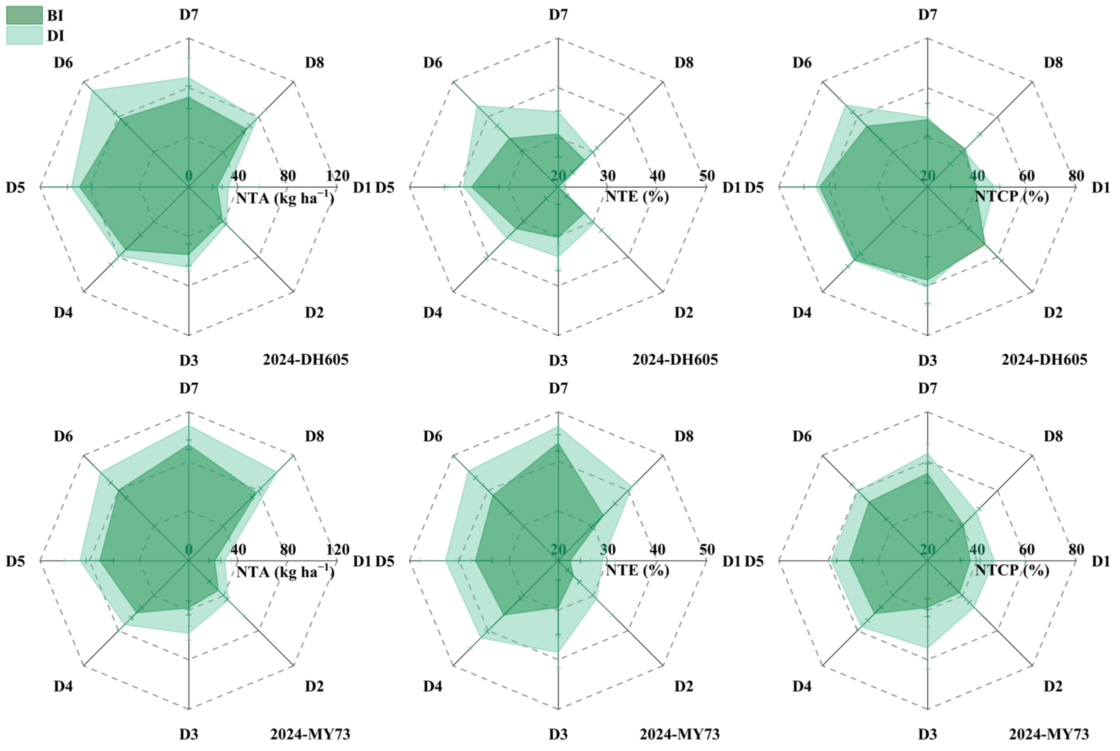

Figure S1. Effects of different water and fertilizer management methods and planting density on nitrogen translocation in summer maize in 2024.

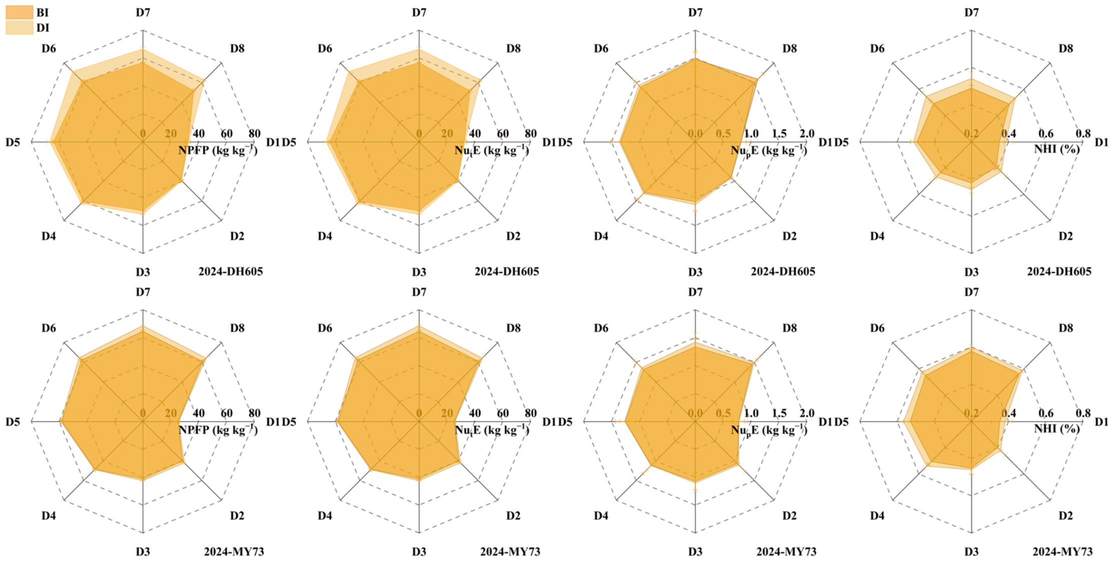

Figure S2. Effects of different water and fertilizer management methods and planting density on nitrogen partial factor productivity, nitrogen use efficiency, nitrogen uptake efficiency, and nitrogen harvest index in summer maize in 2024.
